# Reply to letter to the editor “effect of thrombocytopenia on development of posthemorrhagic hydrocephalus in neonates: few concerns”

**DOI:** 10.1007/s00381-020-04914-x

**Published:** 2020-10-07

**Authors:** Ahmed El Damaty

**Affiliations:** Pediatric Neurosurgery Division, Department of Neurosurgery, Heidelberg University Hospital, Im Neuenheimer Feld 400, 69120 Heidelberg, Germany

Dear Editor in Chief,

We read with great interest the letter to Editor regarding our article “Thrombocytopenia: is it a prognostic factor for development of post-hemorrhagic hydrocephalus in neonates?” and we would like to clarify some points trying to answer the concerns of the authors.

The authors criticize our work in a few aspects, some of which I agree with but others not. They criticized not using a multivariate logistic regression which I agree with but the fact that we have an unbalanced sample size, B1 (37) vs B2 (143), would have shown biased results as the test statistics used are affected by the violation of the homogeneity and normality assumptions of the covariance matrices, in particular from the unbalanced number of observations [1]. We are aiming to continue our documentation and observations to verify our findings in further publications using multivariate analysis.

Their assumption that sepsis could be the cause that patients of group B2 did not survive to develop hydrocephalus could not be proved as we have sepsis described in slightly higher percentage in group B2 (59.5% in B1 vs 71.3% in B2) without statistical significance. Also, mortality percentage was lower in group B2 (27% in B1 vs 20.3% in B2) also without statistical significance. Hence, we do not agree with the assumed explanation of escaping hydrocephalus attributed to higher mortality in group B2.

The prothrombin time, activated thromboplastin time, and thrombin time were reviewed in our cohort, but in our patients, we differentiated between initial thrombocytopenia and secondary thrombocytopenia that occurred later in association with sepsis [2], which was in the majority of cases associated with derangement of the coagulation profile. We agree that the coagulation pathway also plays an important role in determining bleeding tendency, subsequent fibrin deposition, and clot formation, but our theory/assumption was based on the role of TGF-ß1 as a platelet-derived cytokine in the process of scarring and clot firmness.

The detailed information regarding mechanical ventilation and failed extubation were not reviewed in our neurosurgical work. A direct relation of those parameters to posthemorrhagic hydrocephalus could be the focus of interest in upcoming work.

A rapid review in actual literature, for example, Hand et al. [3], denies the association between the occurrence of retinopathy of prematurity and intraventricular hemorrhage, and also the reference used in their letter is relatively an old one (Procianoy et al. 1981) [4].

We reported on the incidence of infection in our cohort and in our study; we excluded patients of suspected post-meningitic hydrocephalus if any.

## References

[1] Ateş C, Kaymaz Ö, Kale HE, Tekindal MA (2019). comparison of test statistics of nonnormal and unbalanced samples for multivariate analysis of variance in terms of type-I error rates. Comput Math Methods Med.

[2] Ree IMC, Fustolo-Gunnink SF, Bekker V, Fijnvandraat KJ, Steggerda SJ, Lopriore E (2017). Thrombocytopenia in neonatal sepsis: incidence, severity and risk factors. PLoS One.

[3] Hand I, Shrier E (2019). Lack of association of intraventricular hemorrhage with retinopathy of prematurity. J Pediatr Neurol.

[4] Rs P, Ja G-P, Hm H (1981). An association between retinopathy of prematurity and intraventricular hemorrhage in very low birth weight infants. Acta Paediatr Scand.

